# Supplementary material for: VHL synthetic lethality screens uncover CBF-β as a negative regulator of STING
Source: bioRxiv. 2024 Sep 6:2024.09.03.610968. Preprint. [Version 1] doi: 10.1101/2024.09.03.610968 (PMC11398426; doi:10.1101/2024.09.03.610968)

bioRxiv preprint doi: https://doi.org/10.1101/2024.09.03.610968; this version posted September 6, 2024. The copyright holder for this preprint (which was not certified by peer review) is the author/funder, who has granted bioRxiv a license to display the preprint in perpetuity. It is made available under aCC-BY 4.0 International license.

Supplementary information

Supplementary Fig. 1. scRNA analysis of type I IFN genes in ccRCC

Extended analysis of single cell transcriptomic data from patients with ccRCC. The average expression and the percentage of cells that express type I interferon genes is shown for the principal cell types within the tumour micro-environment.

Supplementary Fig. 2. Gating strategies for flow cytometry

(**a**-**f**) Representative gating strategies used in flow cytometry experiments in 786O Cas9 cells: competitive growth assay (**a**); cell cycle analysis with BrdU and PI (**b**); cell cycle analysis with PI alone (**c**); SYTOX cell death assays (**d**); Caspase-3/7 Green apoptosis assays (**e**); and staining of γ-H2A.X, and for functional mitochondrial assays (**f**). SSC-A: side scatter (area). FSC-A: forward scatter (area). FSC-W: forward scatter (width).

Supplementary Fig. 1. scRNA analysis of type I IFN genes in ccRCC

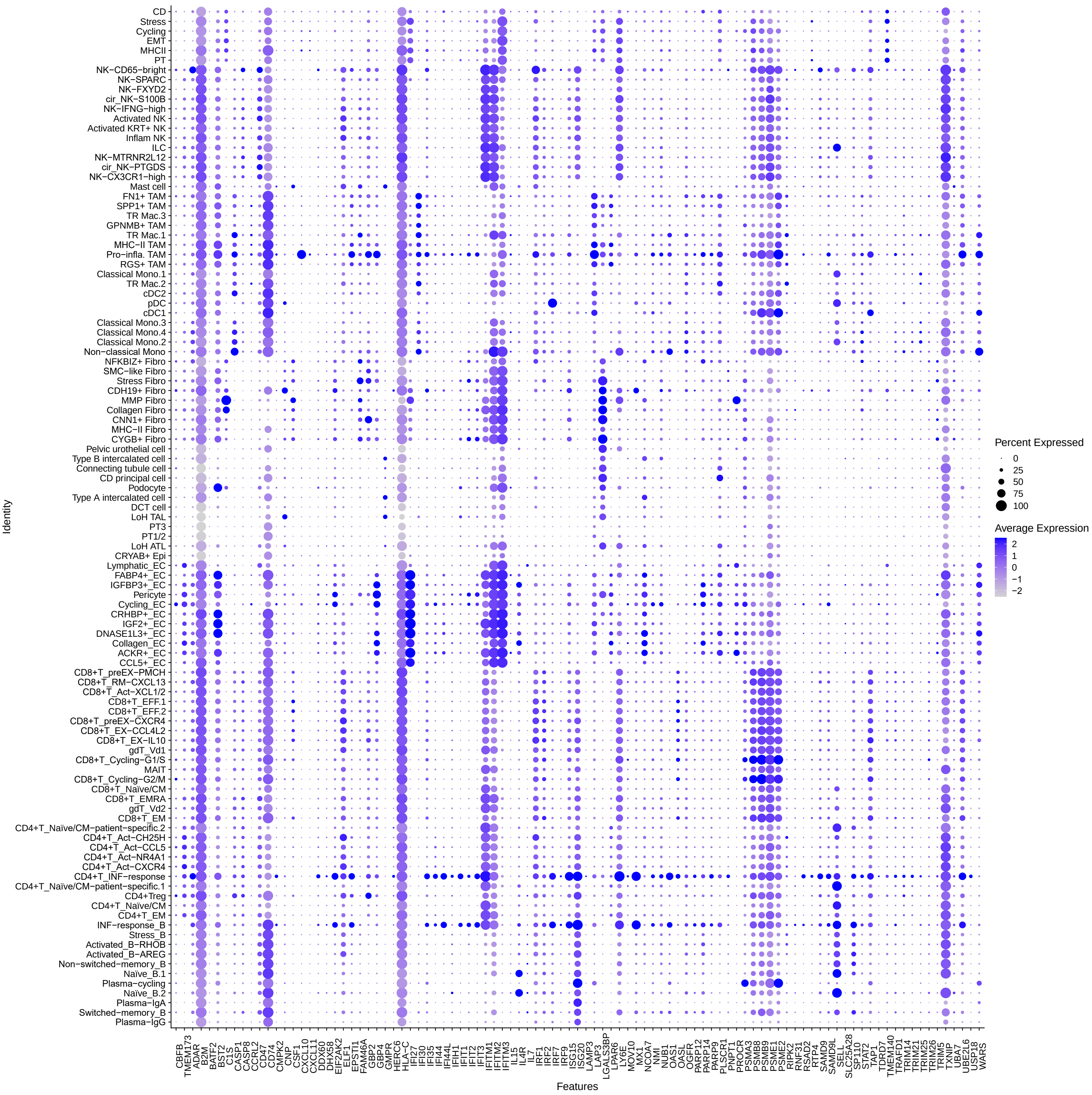

Supplementary Fig. 2. Gating strategies for flow cytometry

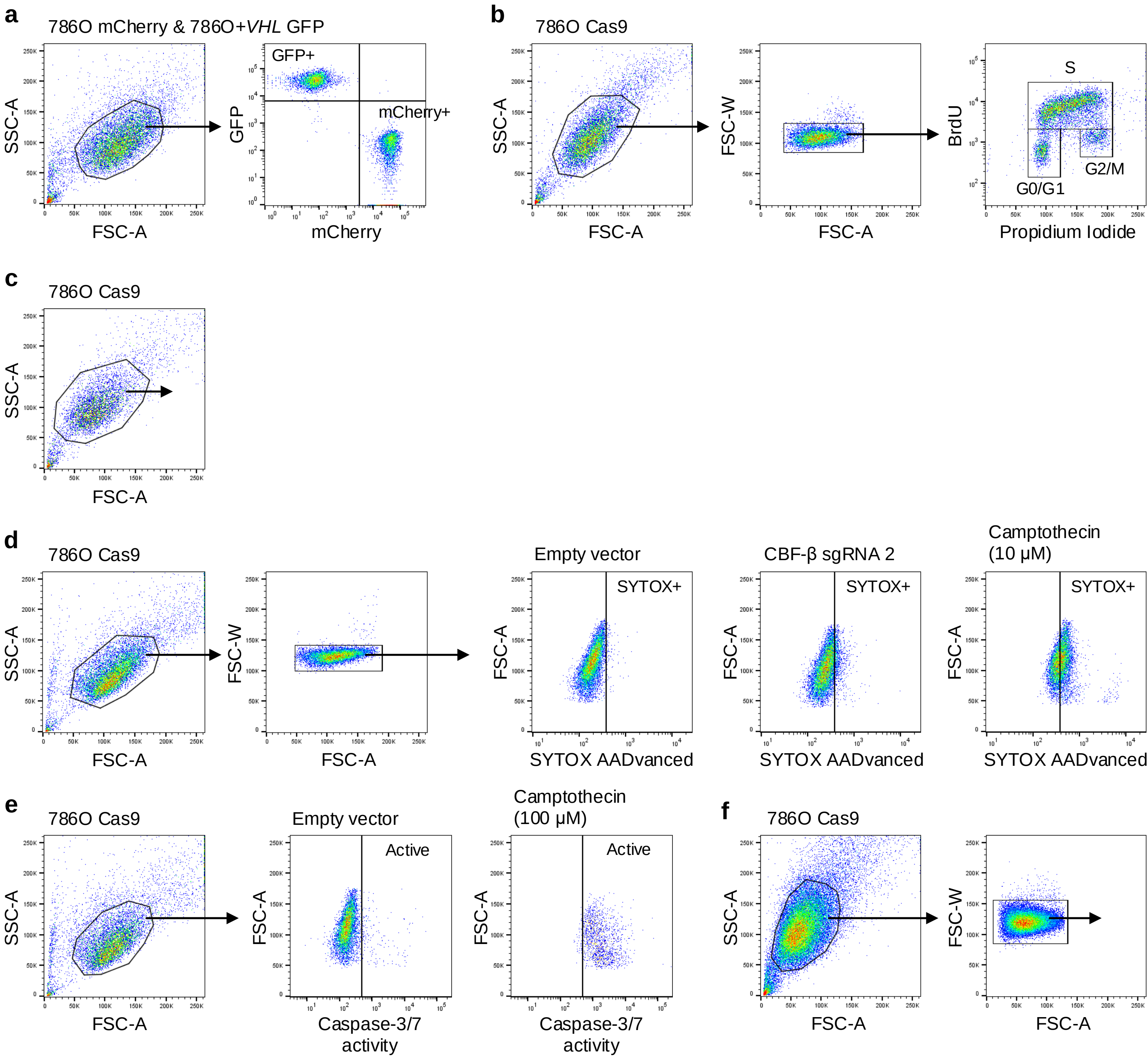

Supplement: Supplement 7 [file NIHPP2024.09.03.610968v1-supplement-7.pdf]
